# Functional near-infrared spectroscopy as a potential objective evaluation technique in neurocognitive disorders after traumatic brain injury

**DOI:** 10.3389/fpsyt.2022.903756

**Published:** 2022-07-22

**Authors:** Fan Chang, Haozhe Li, Ningning Li, Shengyu Zhang, Chao Liu, Qinting Zhang, Weixiong Cai

**Affiliations:** ^1^Shanghai Key Laboratory of Forensic Medicine, Key Lab of Forensic Science, Ministry of Justice, Shanghai Forensic Service Platform, Academy of Forensic Science, Shanghai, China; ^2^Sichuan Provincial Center for Mental Health, Sichuan Academy of Medical Science, Sichuan Provincial People's Hospital, Chengdu, China; ^3^Hongkou Mental Health Center, Shanghai, China

**Keywords:** neurocognitive disorders, traumatic brain injury, executive function, Stroop task, n-back task, fNIRS (functional near infrared spectroscopy)

## Abstract

Most patients with neurocognitive disorders after traumatic brain injury (TBI) show executive dysfunction, in which the pre-frontal cortex (PFC) plays an important role. However, less objective evaluation technique could be used to assess the executive dysfunction in these patients. Functional near-infrared spectroscopy (fNIRS), which is a non-invasive technique, has been widely used in the study of psychiatric disorders, cognitive dysfunction, etc. The present study aimed to explore whether fNIRS could be a technique to assess the damage degree of executive function in patients with neurocognitive disorders after TBI by using the Stroop and N-back tasks in PFC areas. We enrolled 37 patients with neurocognitive disorders after TBI and 60 healthy controls. A 22-channel fNIRS device was used to record HbO during Stroop, 1-back and 2-back tasks. The results showed that patients made significantly more errors and had longer response times than healthy controls. There were statistically significant differences in HbO level variation in bilateral frontopolar, bilateral inferior frontal gyrus and left middle temporal gyrus during Stroop color word consistency tasks and in left frontopolar during Stroop color word inconsistency tasks. During 2-back tasks, there were also statistically significant differences in HbO level variation in bilateral frontopolar, bilateral inferior frontal gyrus, bilateral dorsolateral pre-frontal cortex. According to brain activation maps, the patients exhibited lower but more widespread activation during the 2-back and Stroop color word consistency tasks. The fNIRS could identify executive dysfunction in patients with neurocognitive disorders after TBI by detecting HbO levels, which suggested that fNIRS could be a potential objective evaluation technique in neurocognitive disorders after TBI.

## Introduction

Executive function (EF) refers to the cognitive neural mechanism used by humans in the process of completing a target task (1). As part of cognitive function, EF represents the ability to control the processing of advanced behavior. The impairment of EF leads to neuropsychological defects, such as difficulties in making plans, forming concepts, decision-makings, cognitive flexibility and controlling one's own actions (2). Previous studies have found that the pre-frontal cortex (PFC) plays an important role in EF, and the initial discoveries about EF arose from the exploration of patients with damage to the PFC (3–6). The number of patients with traumatic brain injury (TBI) is increasing yearly for various reasons, such as car accidents and falls. Indeed, it has been found that 77% of patients with craniocerebral injury caused by traffic accidents experience different degrees of neurocognitive disorders, including post-traumatic brain syndrome (PTS) and organic injury (7). Epidemiological evidence indicates that ~14–30% of patients with TBI have varying degrees of cognitive impairment, and ~90% may suffer spontaneous sequelae remission within 2 weeks post-injury, but some impairments may last for several weeks (8). In the weeks to months post-injury, even in mild TBI, some patients still experience symptoms, such as persistent headaches, dizziness, attention deficits, hypomnesia and cognitive impairment, and the most common symptom in patients with TBI is executive dysfunction (9). However, patients tend to focus on the physical trauma and ignore the impairment of EF in the early stages. Furthermore, the TBI patients with cognitive impairments are more likely to develop Alzheimer's disease (AD) (10), which suggests that effective cognitive interventions and imaging tools to identify the patients with impairments in cognitive function, especially mild cognitive impairment (MCI) should be developed.

In recent years, the field of functional near-infrared spectroscopy (fNIRS) has grown (11). The fNIRS is a non-invasive technique with superior temporal and spatial resolution, which provides the real-time measurement of haemodynamic changes in neural regions under the surface of the brain. The fNIRS has been widely applied in the field of cognitive processing, such as in relation to memory (12), attention processing (13), thinking (14), and social cognition (15). Patients with TBI can also be tested using fNIRS to elucidate deficits in memory or attention within the frontal and temporal lobes. According to the literature, selective attention, inhibitory control, memory, and problem-solving are commonly affected in these patients (16, 17). However, the recent literature also highlights contradictory findings regarding brain activation in TBI patients based on various neuroimaging tools, including functional magnetic resonance imaging (fMRI), electroencephalography (EEG), and event-related potentials (ERPs). Khetani et al. (18) examined cerebral activation using fMRI during a working memory task in the first month following pediatric mild TBI and observed that children with pediatric post-concussional syndrome (PPCS) had decreased activation in the posterior cingulate and pre-cuneus during the one-back task. Westfall et al. (19) reported increased brain activation during most difficult working memory load condition in adolescents after TBI. Moreover, using fMRI, Chen et al. (20) reported task-related activation in a subset of regions during a working memory task in symptomatic concussed athletes. Problematically, studies in the field of TBI have mainly focused on deficits in sustained attention or working memory (20–22). A limited number of studies have examined both memory and sustained attention together, but these failed to address changes in neural activity (23). Additionally, previous studies have explored post-injury cognitive function by means of techniques such as ERPs, eye movement (EM), and EEG (12), but these techniques have poor spatial resolution. Therefore, the advantages of fNIRS in the study of cognitive function and its good spatial and temporal resolution warrant further discussion. Moreover, although some researchers have used fNIRS in patients with TBI in the past and focused on the clinical treatment stage, including comparisons during the rehabilitation process. Few studies about fNIRS in neurocognitive disorder after TBI have been conducted when patients seemed to achieve clinical cure, but still suffered persistent executive dysfunction for more than 6 months after TBI.

The study uses a combination of fNIRS with the Stroop and N-back tasks which are considered as effective methods to detect the damage degree of EF in patients with MCI (24). The present study aimed to explore whether fNIRS could be a technique to assess the damage degree of executive function in patients with neurocognitive disorders after TBI by using the Stroop and N-back tasks in PFC areas.

## Materials and methods

### Participants

In total, 60 healthy controls and 37 patients with neurocognitive disorders after TBI were enrolled in the study. Patients were enrolled from September 2019 to November 2020 in the Academy of Forensic Science. Inclusion criteria were as follows: (1) diagnosis of neurocognitive disorder after TBI according to the International Statistical Classification of Diseases and Related Health Problems, Tenth Revision (ICD-10), criterion F07 and Z87.820; (2) mild to moderate traumatic brain injury; (3) conform to the clinical treatment completion criteria 6 months or more after traumatic brain injury; (4) aged between 18 and 60 years old; (5) right-handedness. Mild or moderate injury was defined as an initial Glasgow coma scale (GCS) score between 12 and 15. Patients with a history of chronic disease, mental illness, and substance addictions were excluded. The healthy controls were made up of staff and students in the Academy of Forensic Science. For healthy controls, the following inclusion and exclusion criteria were utilized: (1) aged between 18 and 60 years old; (2) right-handed; (3) no mental disorders; (4) no family history of mental disorders; (5) no organic brain diseases; (6) no alcohol or substance use; (7) no significant medical history; and (8) no use of psychotropic drugs.

The study was approved by the ethics committee of the Academy of Forensic Science. All the methods and procedures of the study were performed in accordance with the criteria laid out by the Declaration of Helsinki and other relevant national and international requirements for human research. All participants signed written informed consent prior to engaging in the study.

### Scales and intelligence quotient (IQ) assessment

Self-designed research forms were used to collect the demographic and clinical characteristics of the participants. The activity of daily living (ADL) scale and social disability screening schedule (SDSS) scale were completed according to the patients' medical records and interviews conducted by the researchers. Patients with total ADL scores >16 are deemed to have impairments in their daily life activities, with higher scores indicating more severe impairment. Patients with total SDSS scores >2 are deemed to have social dysfunction, with higher scores indicating more severe impairment of social function. The Wechsler Intelligence Test (Chinese version) was used to collect the IQ levels of the participants.

### Stroop tasks

The classic color word Stroop experiment was fully computer-controlled and programmed with E-Prime experimental software. Each trial started with the presentation of a red fixation cross for 500 ms and a guide with information about the tasks for 2,000 ms. The Stroop task was revised for our study, to include color word consistency tests and color word inconsistency tests (25). The rest time was used as the baseline. During the task, a color word presented in colored ink was shown on a computer screen. The participants were instructed to press a key (left-handed F when the color word was the same as the ink color or right-handed J when the color word was inconsistent with the ink color) while simultaneously stating the ink color. In color word consistency tests, the color word was the same as the ink color in 91% of the trials. In the color word inconsistency tests, the color word was different from the ink color in 89% of the trials. After the key press action, the word on the screen changed. The two tests were presented in a block design and conducted three times, each with a break period of 30 s between blocks. A red fixation cross was presented on the computer screen during the break period, and the participant was guided to relax and focus on the screen. All responses were digitally recorded.

### N-back tasks

A combined 1-back task and 2-back task were used to quantify working memory (26) in this study. The task was fully computer-controlled and programmed with E-Prime experimental software. Each trial started with the presentation of a red fixation cross for 500 ms and a guide with information about the tasks for 2,000 ms. During the N-back task, a series of stimuli were presented, and the participant was instructed to respond to the stimuli that they saw “N” items-back (1-back or 2-back) (27). In the 1-back condition, the participants were required to memorize the numbers and determine whether the number was the same as the previous one. They were instructed to press the left-handed F key when the number was the same as the previous one, and right-handed J key when the number was different from the previous one. In the 2-back conditions, the participants were instructed to judge whether the number was consistent with the number presented two items before. A 30 s period was inserted between the trials. The stimuli were numbers (1–9), which were pseudo-randomly ordered to ensure that there were no repeats (9–9) and no ordered series (1–2–3). The numbers 1–9 randomly appeared 23 times in the 1-back condition and 25 times in the 2-back condition. As an example, in the 1-back version, the series “1–3–6–3……” may be shown to a participant. For this list of numbers, when they saw 3, they were required to press the right-handed J and when they saw 6, they were required to press the right-handed J, etc. In the 2-back version with the same list of numbers “1–3–6–3 ……,” when the participant saw 6, they were required to press the right-handed J and when they saw 3, they were required to press the left-handed F, always keeping in mind the number presented two numbers previously. All responses were digitally recorded.

### The FNIRS tests

The tests included Stroop or N-back tasks, and each task appeared three times. All participants took part in both types of tasks. No fNIRS data was acquired during the practice phases. At the beginning, the 10 s baselines were used to normalize the data. Additionally, 30 s rest blocks were added between stimuli and during this time, the fNIRS data were saved, and the participants were instructed to rest.

**Figure 1:**
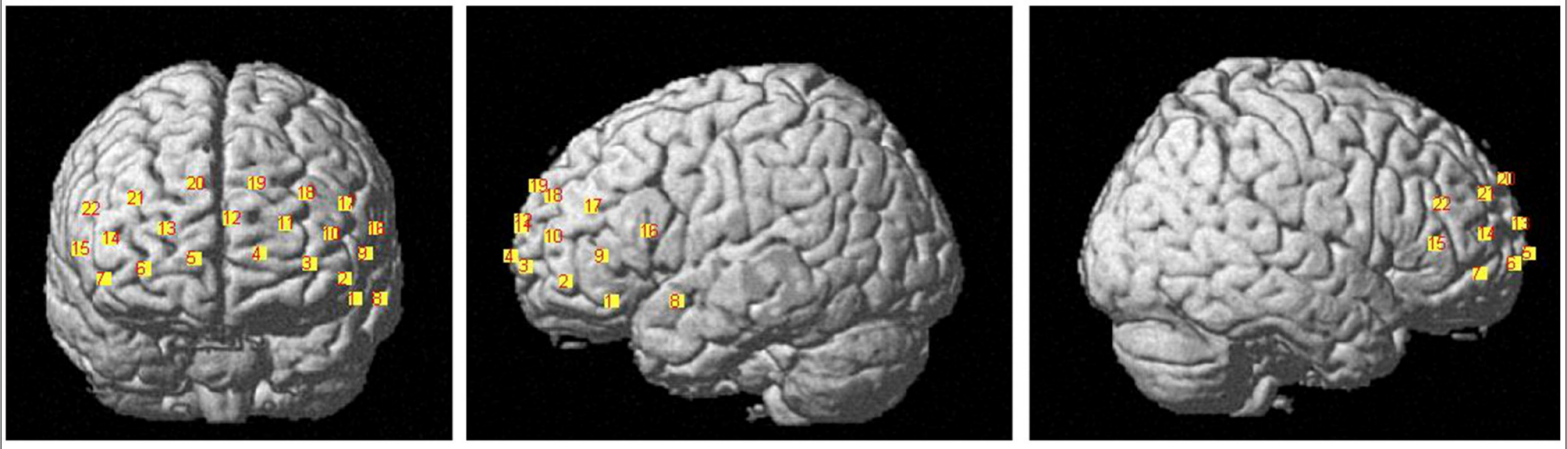
The locations of 22 channels.

The HbO data were acquired using the LABNIRS (Shimazu company, Japan). The 16 probes combined into 22 channels and the 22-channels optical array was placed across the forehead providing bilateral pre-frontal lobe and dorsolateral pre-frontal lobe coverage. The lower side of the probe was flush with the upper side of the eyebrow. The locations of the 22 channels are shown in Figure 1. The FASTRAK three dimensions magnetic digitizer (Polhemus companies in the United States) and the three dimensions position measurement system software (version 4.1.0.0 Shimadzu Corporation, Japan) were used to determine the three-dimensional spatial position of each probe, according to the Montreal Neurological Institute (MNI) reference coordinate system (28).

### Statistical analyzes

The fNIRS data were collected by LABNIRS and pre-processed by denoising algorithms. Heartbeat and respiration noise signals were removed by using a 0.14–0.17 Hz finite impulse response low-pass filter. The instrumental noise and physiological interference from the body were processed by denoising algorithms (29). The concentration change of HbO was calculated by the modified Beer-lambert law. The HbO data were processed and analyzed by NIRS-SPM, a near-infrared data processing package in Matlab software (The MathWorks, USA). Spatial maps were estimated by β values in following general linear model (GLM).


$$\begin{array}{l} Y=X\cdot \beta +\varepsilon \end{array}$$

Y = observed fNIRS data; X = convolution of design matrix and hemodynamic response function; ε = residuals

Spatial maps were created by three contrast-model-matrices in NIRS-SPM. A one sample *t*-test was used to create spatial maps of significant activation for the patients and controls, respectively. Threshold images for the resulting group data used an alpha of 0.05. Areas of significant task-based activity are described in terms of three-dimensional spatial position data.

The data were analyzed by IBM Statistical Product and Service Solutions version 22.0 (IBM SPSS 22.0) and Matlab software. The statistical significance level was set at *P* < 0.05 correction thresholds (corrected by FDR). All data were presented as mean ± standard deviation (SD). Categorical data were compared using chi-square tests. The Student's *t*-test was used to compare the normally distributed variables between patients and healthy controls. The Mann-Whitney *U*-test was used to compare the non-normally distributed variables between patients and healthy controls.

## Results

### Demographic characteristics

A total of 37 patients and 60 healthy controls were enrolled. During the study, four patients and five healthy controls did not complete the tasks. In total, 30 patients and 55 healthy controls completed the study. There were no significant differences between the healthy controls and the patients in age, sex and education. The IQ levels of patients were significantly lower than the healthy controls. The ADL and SDSS scores of patients were significantly higher than the healthy controls (Table 1).

**Table 1:** Demographic and clinical characteristics in patients and controls.

	**Controls** **(*****N*** = **55)**	**Patients** **(*****N*** = **30)**	**Statistic**	***P*** **-value**
Male/female	22/33	16/14	1.729^a^	0.19^a^
Age in years	41.56 ± 10.21	40.89 ± 11.72	0.288	0.78
Education in years	10.53 ± 4.79	10.50 ± 4.46	0.052	0.98
IQ	93.19 ± 19.28	67.33 ± 12.77	9.080	0.001*
GCS	/	14.74 ± 1.28	/	/
SDSS	0.25 ± 0.15	5.83 ± 1.76	−10.160	0.001*
ADL	14.57 ± 1.06	21.84 ± 3.68	−13.162	0.001*

Data were presented as mean ± SD. IQ, intelligence quotient; GCS, Glasgow coma scale; ADL, activity of daily living; SDSS, social disability screening schedule. ^a^chi-square test; other statistics: Student's t-test. *Statistically significant differences.

### Behavioral data during Stroop and N-back tests

The response times of patients were significantly longer than healthy controls during the Stroop color word consistency and color word inconsistency tests (color word consistency: 2,301.03 ± 1,035.62 vs. 1,298.19 ± 559.72 ms, *t* = 5.051, *P* = 0.001; color word inconsistency: 3,150.44 ± 1,241.66 vs. 1,964.98 ± 727.85 ms, *t* = 4.902, *P* = 0.001). The error numbers of the patients were significantly higher than the healthy controls during both the color word consistency and color word inconsistency tests (color word consistency: 10.00 ± 7.59 vs. 3.19 ± 6.74, *t* = 4.125, *P* = 0.001; color word inconsistency: 14.15 ± 9.44 vs. 5.43 ± 8.28, *t* = 4.362, *P* = 0.001).

The response times of both patients and healthy controls were significantly shorter during the color word consistency tests compared to the color word inconsistency tests (patients: *t* = −7.820, *P* = 0.001; controls: *t* = −10.599, *P* = 0.001). Additionally, the error numbers of patients and healthy controls were significantly lower during the color word consistency tests compared to the color word inconsistency tests (patients: *t* = −4.414, *P* = 0.001; controls: *t* = −5.529, *P* = 0.001).

The response times of the patients were significantly longer than the healthy controls during the 1-back and 2-back tests (1-back: 1,825.12 ± 891.01 vs. 1,337.42 ± 904.97 ms, *t* = 2.520, *P* = 0.014; 2-back: 3,074.63 ± 143.65 vs. 2,426.06 ± 1,829.00 ms, *t* = 2.564, *P* = 0.012). The error numbers of the patients were significantly higher than the healthy controls during the 1-back and 2-back tests (1-back: 9.03 ± 6.14 vs. 6.08 ± 4.94, *t* = 3.343, *P* = 0.001; 2-back: 14.19 ± 9.11 vs. 7.10 ± 5.95, *t* = 3.730, *P* = 0.001).

No significant differences in error number between the 1-back and 2-back tests were observed for healthy controls (*t* = −1.903, *P* = 0.056), but the error numbers of patients were significantly lower during the 1-back tests compared to 2-back tests (*t* = −4.632, *P* < 0.001). The response times of both patients and healthy controls were significantly shorter during the 1-back tests compared to 2-back tests (patients: *t* = −6.387, *P* = 0.001; controls: *t* = −7.139, *P* = 0.001).

### The relationships between channels and brain areas

The relationships between channels and brain areas according to the FASTRAK three dimensions magnetic digitizer and the three dimensions position measurement system software were shown in Table 2.

**Table 2:** The relationships between channels and brain areas.

**Channel**	**MNI co-ordinate system**			**Left or right**	**Brodmann area**
	**X**	**Y**	**Z**		
1	−51	31	−13	L	BA47
2	−48	51	−4	L	BA10
3	−34	65	2	L	BA10
4	−15	73	7	L	BA10
5	12	74	5	R	BA10
6	31	68	0	R	BA10
7	49	53	−5	R	BA10
8	−61	7	−12	L	BA21
9	−55	37	6	L	BA45
10	−42	56	13	L	BA10
11	−23	68	18	L	BA10
12	−3	68	21	L	BA10
13	24	70	17	R	BA10
14	46	56	13	R	BA10
15	58	35	7	R	BA45
16	−60	19	15	L	BA45
17	−48	41	26	L	BA46
18	−32	55	30	L	BA10
19	−12	62	34	L	BA9
20	13	64	34	R	BA10
21	35	56	29	R	BA10
22	54	37	24	R	BA46

*MNI, Montreal neurological institute; L, left; R, right; BA, Brodmann area; X axis, sagittal axis; Y axis, vertical axis; Z axis, coronal axis*.

### The FNIRS results during Stroop and N-back tasks

In the Stroop tasks, there were significant differences in ΔHbO levels in channels 3, 4, 8, 9, 11, 12, 14, 15, and 21 between patients and healthy controls during the color word consistency tests. However, there were only significant differences in channels 3, 4, and 12 during the color word inconsistency tests (Table 3). There were no significant differences in other channels during the Stroop tasks.

**Table 3:** The β values during Stroop tasks in patients and healthy controls.

**Tasks**	**Channel**	**Healthy controls**	**Patients**	***P*** **-value**	**FDR**
Color word	3	0.002 ± 0.010	−0.004 ± 0.012	0.041	0.121
inconsistency	4	0.002 ± 0.008	−0.003 ± 0.008	0.014	0.121
	12	0.003 ± 0.007	−0.002 ± 0.010	0.022	0.121
Color word	3	0.003 ± 0.007	−0.003 ± 0.008	0.015	0.085
consistency	4	0.002 ± 0.005	−0.002 ± 0.009	0.031	0.085
	8	0.001 ± 0.007	−0.010 ± 0.002	0.007	0.085
	9	0.001 ± 0.006	−0.011 ± 0.034	0.026	0.085
	11	0.002 ± 0.006	−0.001 ± 0.001	0.031	0.085
	12	0.002 ± 0.006	−0.003 ± 0.007	0.011	0.085
	14	0.001 ± 0.007	−0.022 ± 0.009	0.048	0.113
	15	−0.007 ± 0.009	−0.001 ± 0.019	0.019	0.085
	21	0.003 ± 0.005	−0.001 ± 0.005	0.029	0.085

*Data were presented as mean ± SD. Statistics: Mann-Whitney U-test*.

In the N-back tasks, no significant differences were found between patients and healthy controls during the 1-back tasks. During 2-back tasks, there were significant differences in ΔHbO levels in channels 2, 3, 7, 9, 10, 11, 14, 15, 18, 21, and 22 between patients and healthy controls (Table 4).

**Table 4:** The β values during 2-back tasks in patients and healthy controls.

**Channel**	**Healthy controls**	**Patients**	***P*** **-value**	**FDR**
2	0.002 ± 0.003	−0.006 ± 0.012	0.013	0.047
3	−0.001 ± 0.011	−0.005 ± 0.006	0.005	0.026
7	0.003 ± 0.006	−0.011 ± 0.017	0.001	0.011
9	0.001 ± 0.007	−0.009 ± 0.024	0.001	0.011
10	0.007 ± 0.031	−0.005 ± 0.010	0.021	0.058
11	0.002 ± 0.001	−0.003 ± 0.01	0.028	0.062
14	0.002 ± 0.006	−0.002 ± 0.013	0.028	0.062
15	0.003 ± 0.009	0.019 ± 0.014	0.039	0.077
18	0.003 ± 0.004	−0.003 ± 0.009	0.006	0.026
19	0.001 ± 0.005	−0.005 ± 0.012	0.042	0.077
21	0.003 ± 0.005	−0.004 ± 0.011	0.021	0.058
22	0.008 ± 0.013	−0.004 ± 0.009	0.004	0.026

*Data were presented as mean ± SD. Statistics: Mann-Whitney U-test*.

**Figure 2:**
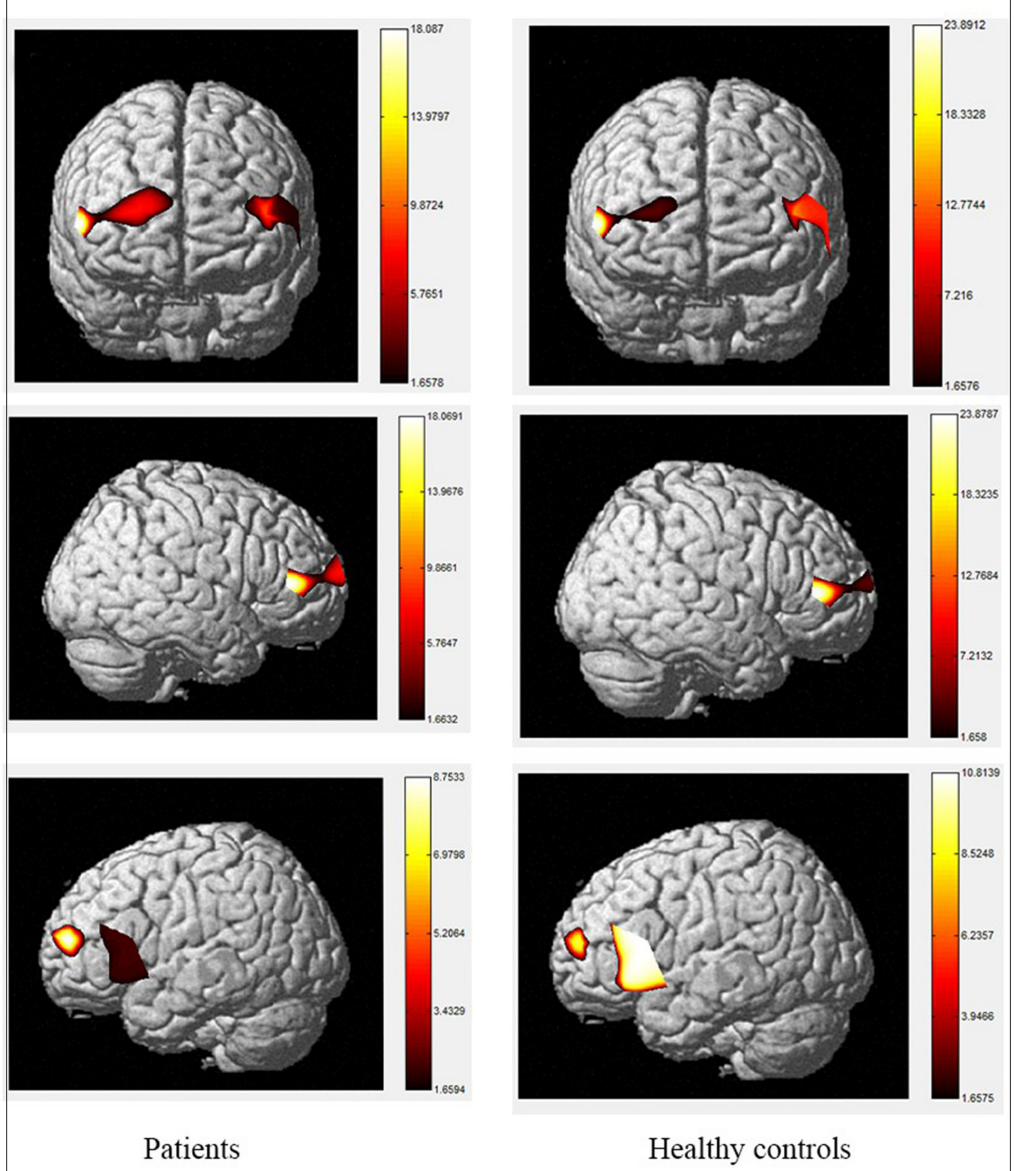
The activation of brain areas during the color word consistency Stroop tasks between patients and healthy controls. The color bars in right sides mean the activated strength of brain areas.

**Figure 3:**
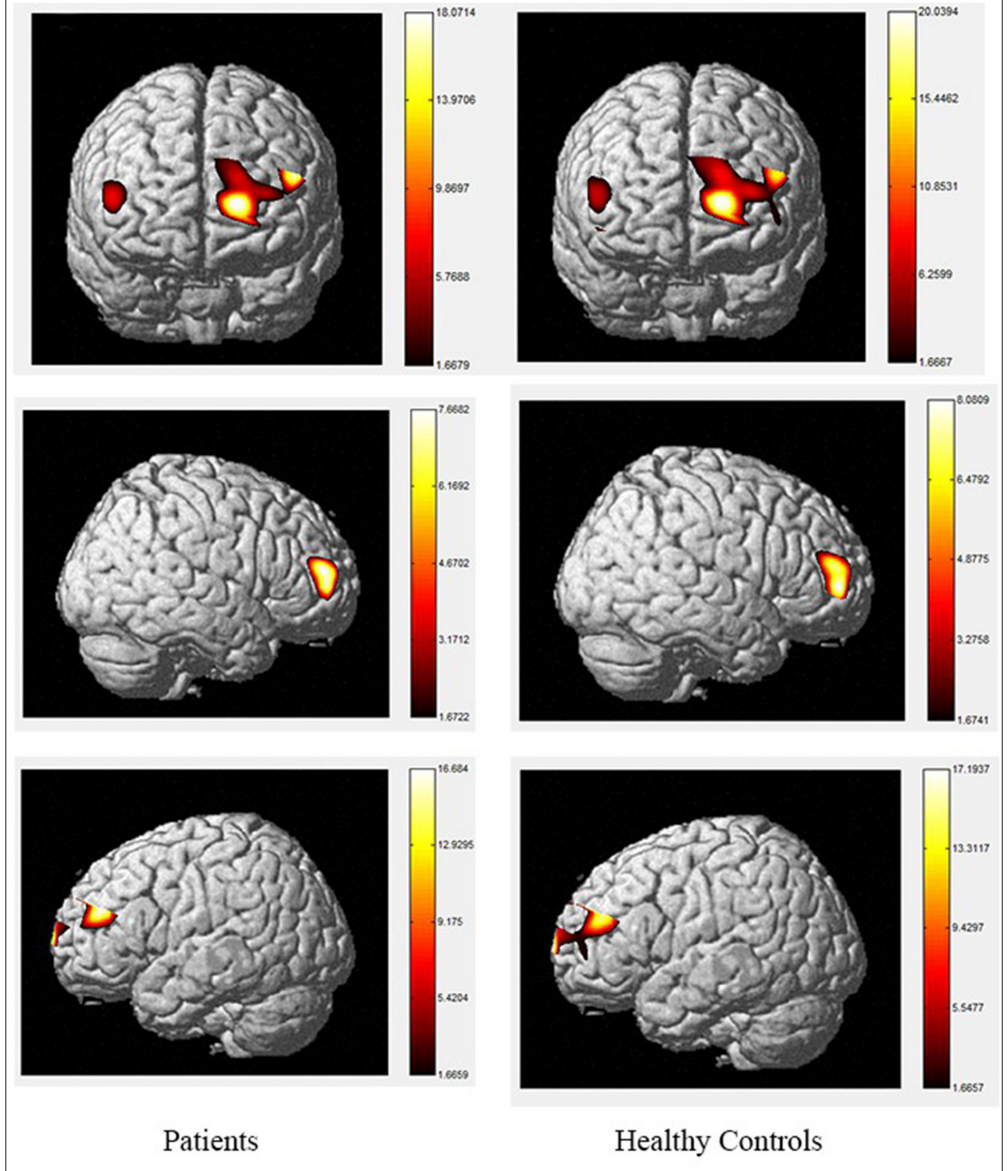
The activation of brain areas during the color word inconsistency Stroop tasks between patients and healthy controls. The color bars in right sides mean the activated strength of brain areas.

**Figure 4:**
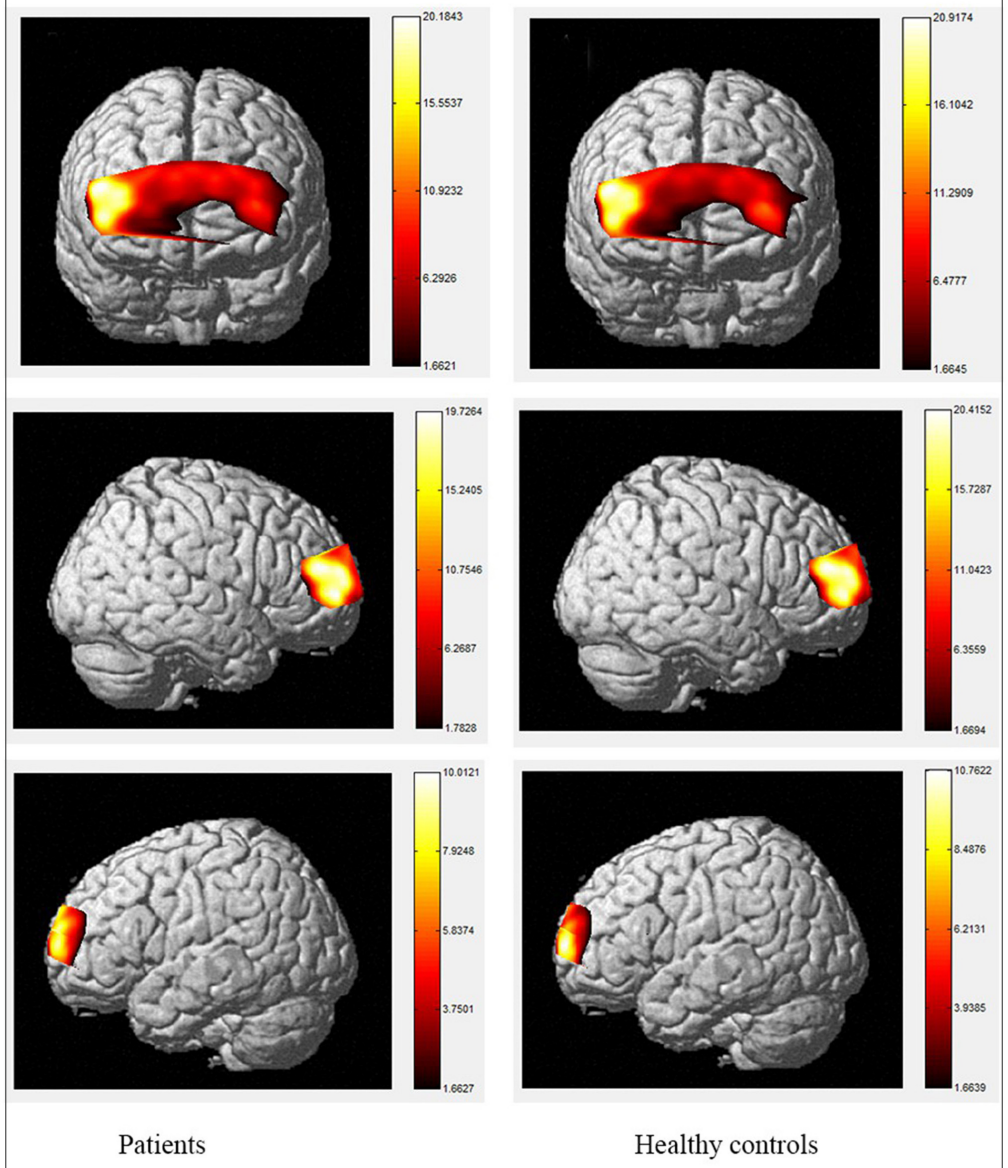
The activation of brain areas during 1-back tasks between patients and healthy controls. The color bars in right sides mean the activated strength of brain areas.

**Figure 5:**
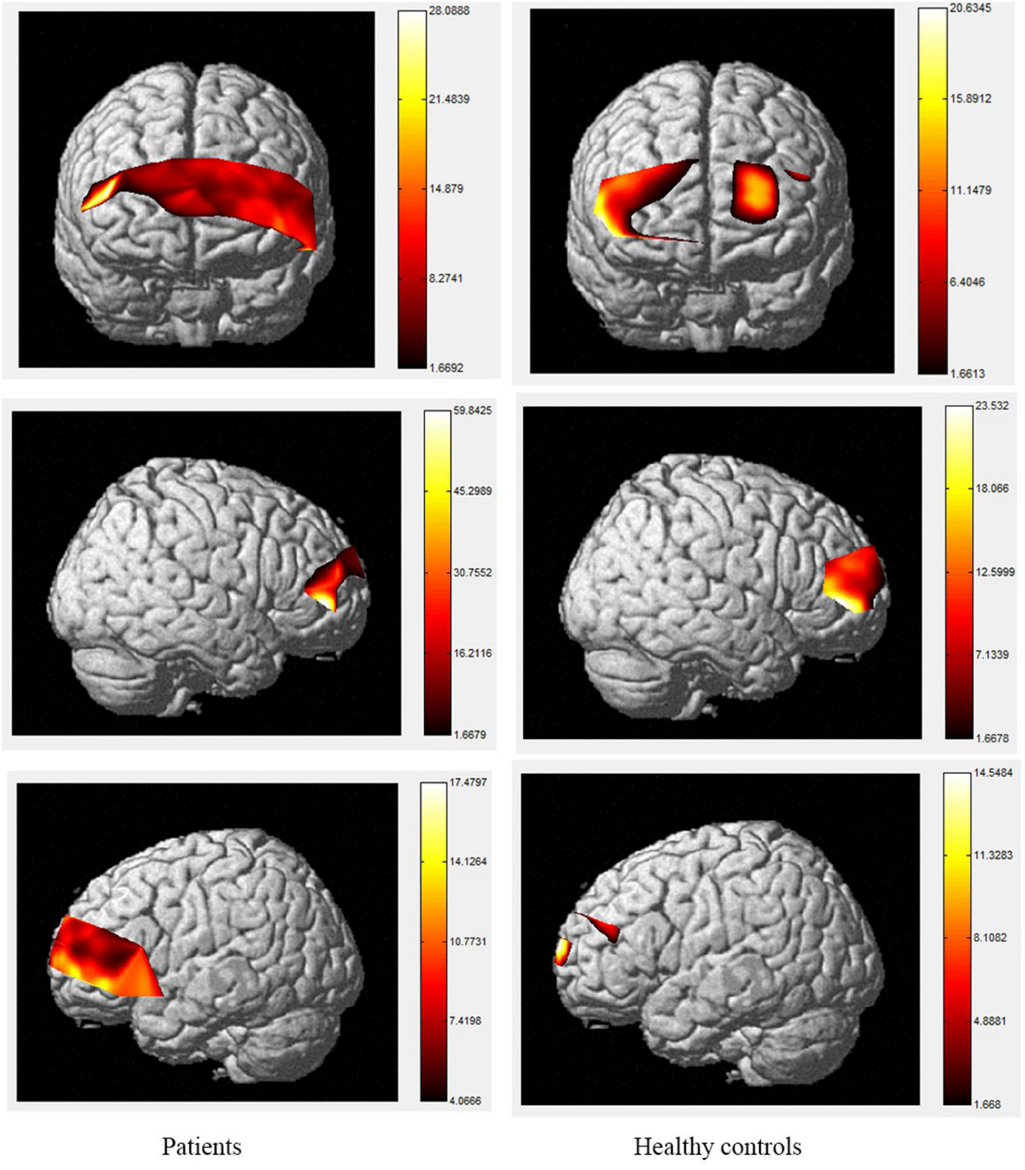
The activation of brain areas during 2-back tasks between patients and healthy controls. The color bars in right sides mean the activated strength of brain areas.

The brain activation maps during Stroop and N-back tasks for patients and healthy controls are shown in Figures 2–5.

## Discussion

Deficits in memory, selective attention, and response inhibition are common after TBI and fMRI studies showed that inefficient pre-frontal lobe function were associated with these deficits (30–32). To address the poor temporal resolution and limitations of fMRI (33), Wijeakumar et al. (34) observed a significant voxel-wise correlation between fNIRS and fMRI measurements, which were applicable to all experimental conditions in the frontal temporal cortex of the brain. The fNIRS is a good technique for studying cognitive function, but the simultaneous evaluation of memory and attention using fNIRS in patients has not previously been performed. This study aimed to address this gap in the literature by assessing changes in cerebral oxygenation, especially HbO, and cognitive accuracy during N-back and Stroop tasks in patients with neurocognitive disorders after TBI using fNIRS.

The fNIRS is a non-invasive monitoring modality, which might be helpful in detecting brain's condition during the acute phase of TBI. Therefore, numerous studies focused on the NIRS-based data and their association with patient functional outcome and a high proportion of studies concluded that fNIRS could be a potential non-invasive technique for assessing TBI (35, 36). However, most studies paid more attention to fNIRS as an continuous monitoring during acute phase of TBI and few studies focused on residual psychiatric symptoms which existed more than 6 months after TBI. The executive dysfunction is a prominent and persistent problem for patients with neurocognitive disorders after TBI, but few fNIRS studies on working memory and sustained attention have been conducted when patients discharged from hospitals for more than 6 months. Meanwhile, fNIRS analysis has not been used to examine changes in the frontal lobes and, especially, frontopolar areas of patients during EF-related N-back and Stroop tasks. Indeed, a study on patients with MCI observed that the combination of Stroop and N-back tasks provided superior resolution for identifying MCI in an elderly group. An initial objective of the study was to identify the differences in memory and sustained attention between patients with persistent symptoms, for whom memory problems were most prominent, and controls using fNIRS (37). The inability to inhibit pre-potent responses and selectively focus on salient information can be measured using the Stroop task, while the N-back task a useful tool for assessing memory. Ultimately, the results revealed significant differences between patients and controls for both behavioral and cerebral oxygenation data during these tasks.

The Stroop and N-back tasks can be modified into two conditions, including simple and more complex, and the subjects are instructed to respond as quickly as possible. These tasks allow us to determine the neural responses to both a simple task and to a more complex task that requires memory, selective attention, and response inhibition. Behaviorally, both the patients and healthy controls exhibited the Stroop effect in this study. Although the 2-back task was complex, and the healthy controls required longer processing time, the number of errors they made was no different from the 1-back task. However, in the patient group, reaction times were longer and the number of errors significantly increased in the 2-back task. A possible explanation for this difference may be that the complexity of the task did not affect the process of storing and processing information in healthy controls. Regardless of the task load, the corresponding neural areas remained effective. Conversely, in the same task, the reaction time of patients was significantly longer and the number of errors was significantly higher, indicating memory and attention impairments in the patient group, which are consistent with previous findings regarding performance in complex executive tasks (38).

Moreover, the patients were limited in complex tasks, and the differences in activated areas increased accordingly. During the 1-back task, there was greater left-sided than right-sided activation in the rostral pre-frontal cortex (rPFC) among controls, and the activation area of the patients was also mainly the rPFC on the left side. Currently, it is believed that working memory includes at least two parts, which are responsible for processing words and spatial information, respectively. Word working memory mainly activates the left hemisphere (dominant hemisphere), whereas spatial working memory mainly activates the right hemisphere. The N-back task is one of the classical models of working memory, and its related brain activation is in line with the neural circuits of working memory, this is relatively consistent with the activation area of word working memory (39, 40) and supports the theory that numbers are also processed as a kind of word information in the left frontal hemisphere. Under the 2-back condition, the activation area of the controls was in the left rPFC and the left dorsolateral pre-frontal cortex (DLPFC). However, the activation area of the patients covered most of the left and right rPFC, in a scattered manner, and the right-side region of the ventrolateral pre-frontal cortex (VLPFC) was also activated. Therefore, the present study demonstrates the involvement of the DLPFC, VLPFC, and rPFC during the 2-back task in TBI patients.

The increased activity during the 2-back task relative to the 1-back task observed in this study is similar to fMRI findings in controls, as tasks with higher memory demands are associated with increased PFC activity (40–47). The spatial locations activated by the 2-back task in this study are also consistent with the areas identified using fMRI, including the bilateral PFC (48, 49). However, our results contradict the studies that reported the anterior cingulate cortex (ACC) was activated during the 2-back task in patients with TBI using fMRI (50–54). A possible explanation for this may be differences in methodology, as fNIRS data cannot be received from subcortical regions such as the ACC, and the number of channels is restricted. In terms of the activation of localized brain regions based on channel differences, both groups exhibited activation in BA46/BA10, including the rPFC, VLPFC, and DLPFC. In contrast, significant differences between the two groups were observed during the 2-back task. Specifically, during this task, the patients exhibited decreased activity compared to controls in channels 2, 3, 9, 10, 11, 14, 18, 19, 21, and 22 which were located in the bilateral DLPFC and rPFC. Previous researches has found that the VLPFC, especially BA44 and BA45, plays an important role in silent reading and retelling, meaning that this area, as well as the parietal cortex, belongs to the phonetic ring and participate in the smooth maintenance of working memory (55, 56). Activation of the bilateral VLPFC was observed during the digital 2-back task in controls, which may reflect neuronal responses generated by extracting stored information and comparing it with current information or may be related to silent reading and retelling. This area was strongly activated in the control group, and we hypothesize that the group had a higher tolerance for silent reading and recitation, allowing them to perform the task better than patients. The differences in these areas suggest that the strategies used by the two groups to complete the task differed significantly.

The color word Stroop test is commonly used to assess selective attention in TBI. The latency difference between the two tasks is referred to as Stroop Interference (SI). In accordance with the present results, previous studies have demonstrated that larger SIs are observed in TBI patients than in healthy controls. The TBI-related increase in SI is typically considered to reflect the decrease in selective-attention after TBI. The current study observed that greater difficulty in both the consistent and inconsistent conditions in TBI patients. These outcomes are contrary to those of Plenger et al. (13), who noted that greater difficulty in color naming after TBI was not reflected in their behavioral data. These differences could have arisen due to the contexts of the paradigm. Indeed, studies using the Stroop paradigm in China have all reported SI in the behavioral data of healthy controls. Furthermore, a recent meta-analysis proposed that TBI-related changes in sensory processing, specifically color vision, could at least partly explain this increase in SI after TBI (57). They proposed that increased difficulty in color vision processing after TBI could be the source of inflated SI, beyond any changes in selective attention. There are several possible explanations for differences in SI results, such as using error rates, lacking mandatory control time, changing the stimuli only after the subject pressed the key, and recording every key response, which may make the task not sensitive enough to gauge small-scale differences in baseline color-naming. During the consistent color condition, the control group activated a large area of the right rPFC, as well as a small area of left rPFC, whereas, among the TBI group, the consistent condition elicited a greater spatial extent of significant activation bilaterally. During the inconsistent task, activation was observed in the same regions in the control and patient groups with the patient group showing a small amount of additional activation in part of the left rPFC, VLPFC and DLPFC. The spatial locations activated by the inconsistent condition in this study are consistent with the regions involved during the Stroop inconsistent task using fMRI, as previous literature has demonstrated significant bilateral rPFC involvement in that task (58, 59). Increased activity in the left hemisphere was expected given the verbalizations required during this task (60). More specifically, activation in the VLPFC was expected given its role in naming and word retrieval, which has been repeatedly demonstrated in various neuroimaging studies (61–65).

Increased activity during the inconsistent condition of the Stroop task compared to the consistent condition in this study is in line with the results of fMRI studies with normal controls, as tasks requiring increased attentional demand are associated with increased pre-frontal cortex activity. Comparisons of the cerebral oxygenation patterns of patients and controls revealed Stroop effects in certain regions of interest (ROIs; BA10, BA46, rPFC, and left DLPFC) for HbO. The accuracy rates of TBI patients were lower than those of controls, and both groups responded more accurately to the consistent condition than the inconsistent condition. These effects support our difficulty manipulation, as the participants made more errors and took longer reflection times in the more difficult inconsistent condition. In addition, the increased difficulty resulted in more response errors and longer response times for the TBIs than for the controls. Moreover, the patient group activated a greater spatial extent, bilaterally extending from the anterior frontal lobes to the left VLPFC regions. Activations in dorsal regions during the simple visual attention task support attention and orientation toward visual stimuli (66–68). Furthermore, between-group analysis of the extent of activity changes from baseline in certain channels (4, 8, 9, 11, 14, 15, and 22, located in BA9, BA21, BA10, and BA45) during consistent condition demonstrates that the patient group had significantly lower HbO responses in bilateral frontal regions during this condition compared controls. These results suggest that patients with TBI generally activate the anterior frontal lobes less efficiently than controls, but the activation was more extensive in the VLPFC, as reported by Plenger et al. (13), who found a similar phenomenon during a dot color-naming task. It is possible that less efficient activity represents damage to an inhibitory area of the brain in the patient group, and more extensive activation reflects neural compensation in these individuals, as a greater degree of processing may be required to accomplish the same task. The fNIRS data supports this sensory theory (65, 69–73). Indeed, in the inconsistent condition, the patients activated the same regions as those active by controls. For controls, there were additional loci of brain activity when performing the color word inconsistent task compared to the color word consistent task; specifically, additional activation was observed in the bilateral PFC.

### Limitations

This study has several limitations. Firstly, the subjects may have demonstrated uncooperative behavior during task execution, thus making examination challenging. Secondly, the sample size was relatively small, which may have affected the statistical power. Thirdly, the study focused on fNIRS as a potential objective evaluation technique in neurocognitive disorders after TBI, and did not investigate the underlying mechanisms. Therefore, larger sample sizes and more mechanism research will be carried out in the.

## Conclusion

In conclusion, based on fNIRS technology, the results of this study find that patients with neurocognitive disorders after TBI show decreased activation strength and increased activation range in brain activation maps during Stroop and n-back tasks, which means these patients may suffer executive dysfunction. Therefore, the fNIRS could be a potential objective evaluation technique in neurocognitive disorders after TBI.

## Data availability statement

The datasets generated for this study are available on request to the corresponding author.

## Ethics statement

The studies involving human participants were reviewed and approved by Academy of Forensic Science. The patients/participants provided their written informed consent to participate in this study. Written informed consent was obtained from the individual(s) for the publication of any potentially identifiable images or data included in this article.

## Author contributions

FC and HL participated in the design of the study and drafted the manuscript. FC, HL, NL, SZ, and CL enrolled patients and healthy volunteers, performed the experiments, and performed statistical analyses. QZ participated in the design of the study and helped with proofreading. WC and HL conceived the study protocol and participated in the design and co-ordination of the study. All authors contributed to the final text and approved it.

## Funding

This study was supported by the National Natural Science Foundation of China (81801881), Science and Technology Committee of Shanghai Municipality (20DZ1200300, 21DZ2270800, and 19DZ2292700).

## Conflict of interest

The authors declare that the research was conducted in the absence of any commercial or financial relationships that could be construed as a potential conflict of interest.

## Publisher's note

All claims expressed in this article are solely those of the authors and do not necessarily represent those of their affiliated organizations, or those of the publisher, the editors and the reviewers. Any product that may be evaluated in this article, or claim that may be made by its manufacturer, is not guaranteed or endorsed by the publisher.
